# PSYCHOSIS AS A MANIFESTATION OF LUPUS AND ANTIPHOSPHOLIPID SYNDROME. ABOUT A CASE.

**DOI:** 10.1192/j.eurpsy.2023.2111

**Published:** 2023-07-19

**Authors:** F. Vílchez Español, R. Carrillo Molina, A. Alcántara Gutiérrez

**Affiliations:** 1 Psiquiatría, Complejo Hospitalario de Jaén, Jaén, Spain

## Abstract

**Introduction:**

A lot of studies have determined the relationship between psychosis and autoimmune diseases. One of the classic examples is systemic lupus erythematosus and antiphospholipid syndrome.

Both are syndromes marked by a state of excessive inflammation and hypercoagulability, respectively. And psychosis is a frequent manifestation of these two diseases, so it is important to take it into account, because psychotic episode triggered by these diseases has a different therapeutic approach from that of primary psychoses.

**Objectives:**

To raise awareness about this fact, we present the clinical case of a 43-year-old woman, diagnosed with systemic lupus erythematosus and antiphospholipid syndrome, who went to the Emergency Department due to agitation and delusional ideation of harm.

**Methods:**

Given that the patient presented a recent altered cranial MRI, the aforementioned pathologies and an acute and poorly systematized clinical onset, we referred her for admission to Internal Medicine due to suspicion of a psycho-organic syndrome of probable autoimmune origin.

**Results:**

After admission to Internal Medicine, corticosteroid treatment was prescribed. After three days, the symptoms remitted and the patient was discharged, starting outpatient follow-up.

**Conclusions:**

It is important not to forget that psychotic symptoms may be due to causes other than merely psychopathological ones, and may belong to other aetiologies and, with it, other therapeutic attitudes.

**Disclosure of Interest:**

None Declared

